# Evaluating the Feasibility and Acceptability of a Culturally Adapted Intervention to Promote Resistance Exercise in Young Black Women: A Randomized Controlled Trial

**DOI:** 10.3390/ijerph23070867

**Published:** 2026-07-03

**Authors:** Chloe S. Jones, Katherine E. Spring, Danielle D. Wadsworth

**Affiliations:** 1 School of Kinesiology, Auburn University, 301 Wire Road, Auburn, AL 36849, USA; katie.spring@pbrc.edu (K.E.S.); wadswdd@auburn.edu (D.D.W.); 2 Department of Social and Behavioral Sciences, Virginia Commonwealth University, 830 E Main Street, Richmond, VA 23219, USA; 3 Ingestive Behavior, Weight Management, and Health Promotion Laboratory, Division of Clinical Science, Pennington Biomedical Research Center, 6400 Perkins Road, Baton Rouge, LA 70808, USA

**Keywords:** resistance training, exercise adherence, cultural adaptations, mHealth, health promotion

## Abstract

**Highlights:**

**Public health relevance—How does this work relate to a public health issue?**

- Improving resistance exercise participation in young Black women addresses rising early cardiometabolic risk in this population.

**Public health significance—Why is this work of significance to public health?**

- To date, there have been limited resistance exercise interventions dedicated towards young Black women. This study addresses a key gap by evaluating culturally adapted behavioral resistance exercise intervention designed to support transition to long-term, independent exercise in young Black women.

**Public health implications—What are the key implications or messages for practitioners, policy makers, and/or researchers in public health?**

- Culturally adapted, supervised resistance exercise programs with ethnically matched trainers can effectively engage young Black women, but sustained participation requires ongoing, tailored support and accountability beyond supervised settings.

**Abstract:**

Young Black women face barriers to exercise and elevated cardiometabolic risk, yet resistance exercise (RE) remains underutilized despite its benefits. We evaluated the feasibility and acceptability of a 24-week culturally adapted RE intervention + text messages in young Black women. Participants were randomized to the motivational exercise group (MEG; *n* = 14) or the standard exercise group (SEG; *n* = 13). Both groups received 10 and 11 weeks of supervised (by a Black woman) and unsupervised RE. MEG received additional cultural adaptations and weekly discussions to build competence, autonomy, and self-regulation strategies + mobile support. Feasibility and acceptability were assessed via recruitment, consent, and retention rates, adherence, and thematic analysis of semi-structured interviews. Recruitment and consent rates were 97.2% and 100.0%, respectively. Retention rates were 93.3% (MEG) and 86.7% (SEG) at 12 weeks, and 93.3% and 80.0% at 24 weeks, respectively. Supervised adherence was 93.9% and 88.8% in MEG and SEG, and 14.3% and 15.4%, respectively, during unsupervised RE. Participants desired continued support and a more tailored mobile experience during unsupervised RE. Supervised RE with ethnically matched trainers was feasible and acceptable. Future interventions should incorporate mobile tools with tailored feedback and accountability strategies to sustain long-term RE to improve health outcomes in this population.

## 1. Introduction

Young adult women between the ages of 18 and 34 years face unique challenges to exercise participation and undergo many life transitions such as moving away from home, getting married, starting new careers, and beginning a family [1,2]. Moreover, this population often reports a lack of time, motivation, and/or support to exercise on a regular basis [3,4]. In addition to the aforementioned barriers, young Black women encounter further obstacles not mentioned by White women such as no role models, lack of knowledge or experience, necessity for childcare [4,5], safety concerns [4,6], facility cost [6], unwillingness to alter their body shape [5,7], fear of inability to exercise due to body size [6], and difficulty sustaining desired hairstyle during exercise [4,8]. These additional self-reported barriers might contribute to the alarmingly high percentage of Black women who do not meet the exercise recommendations (83.5%), which surpasses their White women counterparts (75.7%) [9].

Regular participation in exercise can help decrease the risk for adverse health conditions [10]. This is highly relevant to Black women as they are more likely to have higher rates of obesity [11], hypertension [12] diabetes [13], and cardiovascular mortality [14] compared to their White peers. These disparities often begin early in adulthood as Black adults aged 18–44 years exhibit worse cardiometabolic profiles than White adults of the same age [15], thereby signaling a need for intervention during this age group.

While physical activity guidelines recommend both aerobic and muscle-strengthening activities, achieving adherence to planned resistance exercise (RE), a form of muscle-strengthening activity, introduces distinct behavioral barriers. Unlike lifestyle-based physical activity, planned RE requires structured planning, equipment access, and a foundational understanding of progressive overload, specificity, and proper form and technique. Nationally, women are nearly twice as likely to meet aerobic than muscle-strengthening guidelines (43% vs. 27%, respectively) [16,17], indicating that behavioral barriers to RE adherence are distinct from general physical activity promotion. Despite evidence suggesting RE can result in improved metabolic health [18,19], physical performance and strength [18,20], and body composition [21,22,23], targeted interventions remain scarce. Only eight studies have evaluated muscle-strengthening or RE modes in Black women [24,25,26,27,28,29,30,31]. Amongst these studies, none evaluated short-term (<six months) nor long-term adherence (>six months) to a planned RE regimen [26,27,28,29,30,31], leaving the question of whether its implementation is feasible and acceptable and what strategies are required to support sustained participation.

Theoretical Frameworks such as the Social Cognitive Theory (SCT) and the Self-Determination Theory (SDT) have been shown to increase adherence to physical activity and exercise [32,33,34,35,36], yet target distinct, complementary pathways for behavior change. The SCT provides mechanisms of behavior change through self-regulation skills (e.g., goal-setting, time-management, self-monitoring, eliciting social support, reinforcements, and relapse prevention) to assist with exercise adherence [37]. In contrast, the SDT addresses underlying sources of the motivation that is required to sustain behaviors. Specifically, the SDT states that motivation is steered by the level of self-determinism within an individual and can be increased by fulfilling one’s basic psychological needs (competence, autonomy, relatedness) [38]. Increased feelings of being in control of one’s own actions can lead to higher intrinsic motivation (i.e., internal drive) resulting in higher frequency of the behavior. Thus, SCT-based self-regulation skills can serve as practical methods to enhance perceived competence and autonomy, fostering a shift toward intrinsic motivation. While self-regulation is associated with higher exercise participation in women [33,34,35], how these behavioral strategies drive intrinsic motivation is less understood. The integration of these frameworks is vital for interventions tailored to Black women, where methods aiming to shift motivation intrinsically for exercise has been sparse overall [39,40,41], and even more for RE. Culturally adapting this dual-theory intervention provides an avenue to align practical self-regulation skills with culturally relevant motivational drivers, ultimately optimizing long-term exercise adherence.

Previous exercise interventions for Black women have incorporated culturally adapted strategies including ethnically matched interventionists/trainers [42,43], representative imagery on study materials [44,45], interventions in churches or Black-owned community centers [46,47], highlighting culturally specific barriers [35,45], and social support from family/friends [43,44]. The inclusion of experiences and the social and environmental characteristics of a population gives a sense of familiarity, increasing the acceptability of a behavior [48], and methods have shown efficacy for improving short-term adherence to exercise in Black women. However, due to the lack of long-term follow-up assessments and a primary focus on aerobic exercise [40,41], researchers are unclear if utilizing culturally adapted strategies in conjunction with theoretical frameworks would promote sustained RE.

Lastly, leveraging mobile phones to enhance behavior outcomes could potentially promote program sustainability as it is the most consistently effective avenue to deliver an intervention digitally [49,50]. Data show that 96% of Black adults own a mobile device [51], and nearly 70% Black women are willing to receive text-based health education [52]. Mobile-health (m-Health) exercise interventions in Black women have demonstrated increases in subjective physical activity and exercise outcomes mostly over short-term periods (<3 months) [53,54,55] and one over six months [56]. While short-term mHealth interventions in Black women maintain low attrition rates (2.2% over 6 weeks) [55], longer trials spanning from 4–6 months demonstrated attrition rates between 12% and 35% [54,56]. Notably, existing interventions have focused exclusively on aerobic exercise [57], leaving the efficacy of mobile-based platforms to support structured, planned RE in Black women unexplored.

To address these gaps, Black Women are F.I.R.E. (Fitting in Resistance Exercise) was developed. This intervention uniquely integrated the SCT and SDT frameworks with culturally adapted strategies and mobile support through text messages and phone calls. The purpose of this study was to assess the feasibility and acceptability of a culturally adapted, theory-based 24-week RE intervention designed to support sustained RE adherence in young Black women.

## 2. Materials and Methods

### 2.1. Study Design

**Figure 1:**
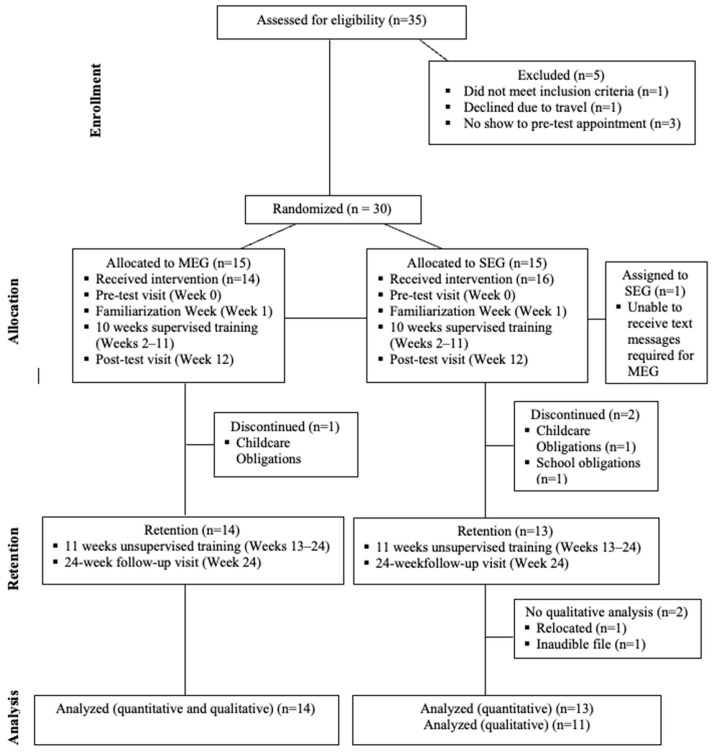
CONSORT Diagram and Study Timeline.

This study was a randomized controlled trial design (#NCT05733260: 16 December 2022). Study procedures were conducted and reported on the basis of the Consolidated Standards of Reporting Trials (CONSORT) 2010 statement. A flow diagram of the trial is shown in Figure 1. Women were recruited for a 24-week study arranged in the following manner: Week 0: pre-test visit; Week 1: familiarization week; Weeks 2–11: supervised RE; Week 12: post-test visit; Weeks 13–23: unsupervised RE; Week 24: 24-week follow-up visit. Pre-, post-, and follow-up testing occurred in a university laboratory at Week 0, Week 12, and Week 24, while the supervised training sessions took place in a community fitness center. All laboratory visits to campus consisted of similar procedures except for administering the informed consent and demographics survey in the pre-test visit and semi-structured exit interviews at the 12-week post-test and 24-week follow-up.

At the pre-test visit, following consent, assessments included completion of psychological and behavioral surveys (Basic Psychological Needs in Exercise Scale, Behavioral Regulation in Exercise Questionnaire-3, Self-Efficacy for Exercise scale, and the Physical Activity Self-Regulation-12), seated blood pressure, a fasted blood finger stick (blood glucose and lipid profile), height, weight, waist circumference, body composition scan using the dual energy X-ray absorptiometry, and an upper and lower body 3-repetion max test. During the pre-test visit, women were randomized in a 1:1 ratio to either the motivational exercise intervention group (MEG) or the standard exercise group (SEG) determined by a coin flip performed by a member of the research team. Because of the behavioral nature of the intervention, blinding of participants and intervention staff was not feasible. Participants were aware of the intervention components outlined within the consent form, and trainers were required to deliver group-specific content. Therefore, neither participants nor interventionists were blinded to treatment allocation. However, research staff responsible for conducting outcome assessments were blinded to participant group assignment to minimize potential measurement bias and all participants completed enrollment and baseline assessments prior to randomization, minimizing the potential for selection bias during recruitment and assessment procedures. Standardized intervention protocols and procedures were used to promote consistency in intervention delivery across participants.

At the pre-test visit, women were registered as a member of an off-campus community fitness center in which they received a complimentary, life-long membership valued at $30. Following, women completed RE twice per week, one-on-one with a trainer, and 11 weeks of unsupervised RE after the 12-week post-test visit. At the post-test and 24-week follow-up, visits included a brief semi-structured exit interview, and $25 was provided to the women after the 24-week follow-up visit for completion of the study. All procedures were approved by the Auburn University Institutional Review Board for Research (protocol #18-323 AR 1809).

### 2.2. Participants and Recruitment

Women between the ages of 18–34 years who self-identified as a Black woman and were non-exercisers were targeted recruits for the study. A non-exerciser was defined as someone who participated in exercise less than three times per week for 30 min for the past three months. Other eligibility requirements included being non-pregnant or planning a pregnancy and a resident or employee of Auburn, AL. Residence or employment status was required for the community fitness center membership. Women were considered ineligible if they were unable to commit to two non-consecutive days of supervised RE.

Recruitment strategies consisted of disseminating digital and physical flyers. Digital flyers were sent through email to past Kinesiology study participants, presidents/secretaries of student and faculty/staff organizations, Black faculty/staff at the university, and through student group chats (e.g., Groupme). Physical copies of the flyers were distributed in-person at Black student organization meetings and hung in community and student residential settings in the city including the following settings: daycare centers, community centers, mailbox rooms and bus stops in student complexes. Additionally, at the in-person student meetings, the PI, who identifies as a Black woman, provided an overview of the study and the importance of increasing healthy behaviors as a Black woman. Interested participants underwent a telephone screening prior to the pre-test visit to ensure they met the eligibility requirements listed above as well as confirmation of being at low risk for exercise determined by the Physical Activity Readiness Questionnaire [58]. All eligible women were then scheduled for a pre-test visit at the on-campus laboratory.

### 2.3. Resistance Exercise Protocol

All participants engaged in two days of RE during supervised training sessions. Supervised RE consisted of two alternating protocols (Protocol A and Protocol B) with seven full-body exercises using a combination of free weights and machines available at the community fitness center. Each session lasted approximately 60 min with 10 min dedicated to a dynamic warm-up and a cool-down including static stretching. Rest periods between sets were not measured. During the familiarization week (Week 1), women completed all exercises of both protocols (14 exercises total) for 12 reps, two days during that week. Trainers demonstrated and focused on proper form, technique, and safety for all exercises. Starting Week 2, women trained at a moderate intensity (60%) based on their 3-repetition max test for upper and lower body and used a linear progression approach at the following training volumes across the 10 weeks: Week 2: 2 × 12, Weeks 3–5: 3 × 12, Weeks 6–8: 3 × 10, and Weeks 9–11: 3 × 8. During unsupervised exercise, women were encouraged to continue the same protocols at the same time but at their preferred gym location twice per week. As the primary outcome for this study was feasibility and acceptability of the program, women were not required to report training volume during the unsupervised phase. Participants were restricted to completing the RE program provided by the research team during the supervised portion of the study due to the assessment of cardiometabolic risk factors as secondary outcomes. Other forms of exercise were permitted during the unsupervised phase.

### 2.4. Culturally Adapted Methods

Both groups completed their weekly training sessions at a local community fitness center in a predominantly Black neighborhood consisting of predominantly Black staff. Women were also matched and trained by a Black woman trainer. Women in MEG received additional cultural adaptations outlined below.

### 2.5. Motivational Exercise Group

Participants randomized to the MEG intervention were trained one-on-one by the same trainer. During sessions, participants received an infographic with depictions of Black women exercising containing information related to exercise education, barriers and motivators for exercise, and theory-based concepts such as competence, autonomy, and self-regulation. The topics on the infographic were discussed in detail twice per week during their supervised sessions with their trainer between sets of exercises starting after the warm-up session.

Exercise education materials aimed to increase participants’ competence related to exercise and focused on the national physical activity recommendations for aerobic and muscle-strengthening physical activity, basic resistance training guidelines (e.g., progressive overload and specificity), and the benefits of exercise participation. Barriers and motivators were informed by other Black women who reported their experiences with exercise in the literature. Self-regulation strategies to help increase exercise included encouraging participants to set goals, self-monitor their exercise, be cognizant of time management, seek social support, introducing reinforcements, and strategies to prevent relapse in their behavior. An overview of the weekly topics and the targeted theoretical concept can be found in Table 1.

To support an autonomous regimen, participants in MEG had the option to choose the protocol they wanted to complete on day 1 of the week (A or B) with the remaining protocol to be completed on day 2. Other autonomy-supportive strategies in MEG included discussion and reflection emphasizing participants’ control of their exercise journey including reasons for exercising, the types of exercise they choose (post-study), and when and where they engage in them.

Lastly, women in MEG received automated twice weekly text messages to reiterate the weekly topics discussed in-person over the 10-week period of supervised training. During unsupervised training, text message frequency was reduced to biweekly and were more motivational to encourage ongoing participation. In addition, women received two telephone calls during the unsupervised training at Week 16 and Week 20 discussing facilitators and barriers to their current exercise behaviors, and how to address challenges.

### 2.6. Standard Exercise Group

Participants in SEG were trained one-on-one by different trainers, in which these trainers were only in contact with the women in SEG. Participants remained with the same trainer over the course of the 10 weeks, except in cases where unforeseen emergencies required last-minute substitutions. Women in the SEG completed the same RE protocol as those in MEG. Other than education on how to properly and safely complete an exercise (Week 1 information in Table 1), no further exercise education (e.g., topics in Week 2 of Table 1) were provided to avoid possible increases in competence related to RE. SEG did not receive any motivational strategies or autonomy support. Women completed Protocol A on day 1 for the first five weeks, and Protocol B on day 1 the second five weeks. Trainers for the women in SEG were discouraged from speaking to participants about deviating plans of RE (unless physically necessary) and were instructed to strictly follow the protocols of the study design.

### 2.7. Study Outcomes

Although secondary outcomes were measured in this study (psychological, behavioral, and cardiometabolic outcomes), this manuscript focused on the primary outcomes regarding feasibility and acceptability. Feasibility was assessed by recruitment rate, consent rate, retention, measurement tools, adherence to supervised and unsupervised RE, and number of completed phone calls during unsupervised RE (MEG only). An analysis in G power calculated an a priori suggested sample size of 24 participants (12 per group) with an effect size of 0.50, alpha level of 0.05 and power of 0.80. The moderate effect of 0.50 was chosen based on the effect of RE on outcomes variables (cardiometabolic and body composition) calculated from several meta-analyses [19,20,59]. Therefore, the study aimed to recruit 36 participants, with a 20% oversample in case of attrition.

Recruitment rate was defined as the number of interested participants divided by the target recruitment goal. Consent rate was determined by the number of participants who consented to the study divided by the number of eligible participants who did not drop out prior to their pre-test visit. Retention at 12-week post-test and 24-week follow-up was defined as number of participants who completed assessments at each timepoint out of the total consented participants who were randomized to each arm. Feasibility of measurement tools consisted of an assessment of the amount of missing data from questionnaires and surveys at in-person testing visits and completion of surveys during unsupervised RE (inquiry about RE frequency). Adherence to supervised RE was calculated by the number of completed supervised sessions divided by the total number of sessions (20 sessions). During unsupervised RE, participants self-reported the number of days of RE over the past weeks at Weeks 18 and 24 via survey. Unsupervised adherence was evaluated by the percent of women who completed at least two days of RE per week during the unsupervised portion of the study (22 sessions minimum). Lastly, completion of scheduled check-in calls (MEG) and the number of required study personnel for data collection were evaluated.

Acceptability was assessed via brief semi-structured interviews at Week 12 and Week 24 by exploring participants’ perception and experience with supervised and unsupervised RE. Interviews at Week 12 were centered around the following domains: reasons for joining the study, overall experience with the study and their trainer, viewpoints on RE before and after the study, strengths and challenges, and plans for future exercise. Interviews at Week 24 focused on topic areas such as participants’ transition from supervised to unsupervised RE, barriers and facilitators for RE, and future participation with RE. Women in MEG at both timepoints additionally discussed their experience with the weekly in-person discussions, text messages, and mobile phone calls both while supervised and unsupervised. Interviews were conducted by the authors DDW and KES, neither of whom were trainers for intervention.

### 2.8. Statistical Analysis

Means, standard deviations, and frequencies were calculated for all demographic variables and quantitative study outcomes. A multivariate analysis of variance (MANOVA) assessed the differences in demographic variables between the two groups at baseline, and statistical significance was set at *p* ≤ 0.05. All quantitative analyses were performed using IBM SPSS version 27 (IBM Corporation: Armonk, NY, USA). Responses to the open-ended questions during the semi-structured interviews underwent a thematic analysis by the PI and a secondary investigator (DDW). Inductive coding was used to create codes based upon patterns and themes found within the data [60]. This analysis included multiple rounds of coding with the first round involving investigators individually reading each of the interviews to familiarize and interpret the context of the scripts. Next, authors assigned key words or codes to phrases of the transcripts that reflected a concept or theme. Once an initial round of coding was complete, the investigators met to discuss the codes that were developed independently along with themes, categories, and subcategories based on their findings [60]. During this time, the investigators deliberated about discrepancies in codes and some codes were renamed or merged to most accurately reflect the context of the transcripts. At the end of the meeting, overall themes and a final codebook was developed. This codebook was used and applied to all transcripts to ensure the capturing of all concepts to accurately represent the experiences of the participants.

## 3. Results

Thirty-five women showed interest in participating in the study. The majority of women were recruited via Kinesiology or university contacts (*n* = 8), undergraduate Black student organizations (*n* = 8), Black graduate student organizations (*n* = 7), and word of mouth (*n* = 7). Other sources of recruitment included campus flyers (*n* = 3) and community/apartment flyers (*n* = 2). This study required data collection amongst two cohorts, therefore recruitment took place for three weeks in February and August of 2023. After women underwent the telephone screening, two women were deemed ineligible due to frequent exercise participation and scheduling conflicts. Additionally, three eligible participants did not attend their pre-test visit and were unresponsive to follow-up contact.

Ultimately, 30 women were eligible to participate in the study and were randomized to either MEG or SEG. At Week 2, one participant in MEG was reassigned to SEG during due to the inability to receive the essential weekly text messages, which was a requirement for MEG. Throughout the intervention three women withdrew from the study reporting childcare (*n* = 2) and school commitments (*n* = 1). Notably, two of the women that withdrew from the study had four children each, setting them apart from the rest of the participants that reported having no children. Therefore, 27 women completed the study (MEG: *n* = 14; SEG: *n* = 13) and participant demographics can be found in Table 2. With the exclusion of age, no significant differences were determined between the two groups at baseline in terms of demographic information. Post-test visits occurred within two weeks after the final in-person training session in May and November of 2023. Final 24-week visits occurred over a span of two weeks in August of 2023 and February of 2024.

### 3.1. Feasibility Outcomes

This study was able to recruit 35 women who showed interest, yielding a 97.2% recruitment rate based on the targeted number (*n* = 36). Of the women who were eligible for the study (*n* = 30), 100.0% consented. Retention rates for the sample were 90.0% at 12-week post-test, and 86.7% at 24-week follow-up. Retention rates in MEG were 93.3% at 12-week post-test (14/15), and 93.3% at 24-week follow-up (14/15). In SEG, retention rates were 86.7% at 12-week post-test (13/15) and 80.0% at 24-week follow-up (12/15). Questionnaire completion rates were 100.0%, 99.9%, and 99.9% based on surveys completed at the pre-, post-, and 24-week follow-up visits, respectively, for all participants. Supervised adherence rates were 93.9% in MEG and 88.8% in SEG. Unsupervised adherence rates were 14.3% for MEG and 15.4% in SEG who adhered to two or more days of RE. More women in MEG completed at least one or more days of RE during the unsupervised period (64.3%) versus the women in SEG (38.5%).

During unsupervised RE, 100.0% of the women responded to the survey inquiring number of days of RE at checkpoint 1 (18 weeks) and 96.2% responded at checkpoint 2 (24 weeks). Women in MEG completed 100.0% of the first check-in call compared to 85.7% for the second check-in call during unsupervised RE. Two women did not respond to phone calls after two attempted scheduled calls. Lastly, testing visits required a minimum of two study personnel, and supervised RE sessions required a total of three trainers (one for MEG, two for SEG).

### 3.2. Acceptability Outcomes

Brief semi-structured interviews averaged 7.82 min at 12-week post-test and 4.68 min at 24-week follow-up. In MEG, 14 women completed both 12-week and 24-week follow-up interview sessions. In SEG, 13 women completed their 12-week interviews, and 12 completed the 24-week follow-up interviews. In this group, one participant relocated and one participant’s interview was inaudible due to technological issues with the recording thereby leaving 11 interviews to analyze in SEG at 24 weeks. Three themes emerged from analysis of the semi-structured interviews at 12-week post-test and 24-week follow-up which were (1) Pre-study Perceptions, (2) Experience with Supervised RE, and (3) Experience with Unsupervised RE. Qualitative results were the same in both groups during supervised RE, but differed during unsupervised RE. All themes, categories, and subcategories can be found in Table 3.

### 3.3. Theme 1: Pre-Study Perceptions

Women in both groups expressed their excitement to join the study due to it being geared towards Black women as some stated this was a rare opportunity in the city they live: “Being specific towards Black females made it more personal. I felt like it might be a good thing to do especially since we’re more prone to diseases and stuff,” (P9 SEG). Having a Black woman as a trainer was also a determining factor as participants felt they would be more at ease starting an unfamiliar program with someone who shared and understood their cultural experiences. A woman in MEG stated, “That definitely influenced my decision … She’ll understand [things] that maybe a man or a White woman may not understand,” (P4 MEG).

Living an overall healthier lifestyle was also on the forefront of the women’s minds. Women discussed needing a kickstart to begin exercising and also mentioned their interest in learning how to perform RE: “I was really looking for an opportunity to try to be healthier and start exercising, and I really wanted to lift,” (P1 SEG). Resistance exercise was unfamiliar to many of the participants and they spoke of being afraid to perform RE due to little to no knowledge and fear of injury, “It was kind of intimidating because it’s weights and unlike cardio, you could really hurt yourself,” (P13 MEG). Another woman stated, “I’ve always been kind of interested in resistance training, but I’ve never known how to get started,” (P14 MEG). Other apprehensions about performing RE centered around the belief that these exercises were only for body builders or professional athletes. Women feared RE would make their body appear bulky or result in a manly figure: “On Instagram, and Tik Tok and stuff we see people that are weightlifters and they’re kind of big and buff and I’m like, I don’t want to look like a man,” (P3 SEG).

### 3.4. Theme 2: Experience with Supervised Resistance Exercise

Women described several mental benefits related to performing RE such as feeling accomplished, being in an uplifted mood, feelings of enjoyment, and/or that it provided a space to relieve their stress, “I would feel nice right afterwards. I felt like I was mentally clear and could like tackle the day,” (P1 SEG). A participant in MEG also said, “I think that my moods increased and I looked forward to coming to the workouts because it was nice,” (P28 MEG). Women perceived the increases in weight for the exercises as a challenge that they wanted to conquer. Despite initial feelings of intimidation, RE became less daunting as the study progressed. Women reported increases in their knowledge and confidence to perform RE and eventually they viewed it as something that was attainable. Participants made comments such as, “I think of it as a challenge. Every time I go up in weight, I’m like, ‘okay, that wasn’t so bad’,” (P21 SEG), and “After having done it, I know that it’s not as intimidating as it seems. Even though it’s a bit more complicated than just like running,” (P13 MEG).

Study participants had a strong appreciation for the community-based gym facility that provided a small, intimate, and comfortable setting for novice resistance exercisers with light traffic: “I don’t like being around a lot of people when I’m exercising. It feels like people are watching me so going to the [study’s fitness facility] where it’s so small… I’m comfortable working out there,” (P9 SEG).

Participants liked being supervised by a trainer as it held them more accountable and committed to their in-person sessions and the research study. A woman in SEG stated, “She showed up for me… the accountability piece. I’m there because someone’s expecting me,” (P15 SEG). Occasionally, work, school, and life obligations altered the women’s schedules therefore they were pleased to have trainers willing and able to flex their typical workout session for a more convenient time: “There were some days where I wasn’t able to come at the time that I picked and she was still able to accommodate me,” (P14 MEG).

Women spoke highly of all the trainers and felt that they were relatable, encouraging, and in some cases, like they gained a new friend or formed a sisterhood. Participants expressed feeling like they were in a safe and welcoming space for Black women which made them comfortable and motivated throughout the study. One participant commented, “It’s kind of like a big sister. It was really nice that it wasn’t just strictly lifting and we’d be able to talk about our days and stuff,” and, “[She was] very warming and welcoming. It was like I already knew her,” (P17 SEG).

Women randomized to MEG discussed their liking of the weekly topics and preference for discussing them in-person versus text messages. Conversations in-person felt more tailored for the participants and gave them opportunities to ask follow-up questions or for recommendations. Although some participants stated they liked the text messages in conjunction with the in-person talks, others stated they barely read the texts. A couple of differing statements made by the women in MEG were “It’s nice because they’re right there and you can just ask questions. The automated text, I don’t think it’s built to reply to it with any follow up,” (P28 MEG) and, “I can look back on them to remember what we talked about, which is helpful because I’m forgetful” (P22 MEG).

### 3.5. Theme 3: Experience with Unsupervised Resistance Exercise

During the unsupervised portion of the study, women in MEG reported performing RE on one or more days of the week with the addition of cardio such as walking or group exercise classes. After receiving two phone calls during the unsupervised period, women stated they were more motivated to complete their RE routines if they had stopped or became less consistent: “Once I got off the call with her, I was like ok I need to get back. That’s really where I [would] spring back into…maybe instead of once a week, it’d be more like two times a week,” (P2 MEG). Despite efforts to promote continual exercise, many women were not able to commit to two days of RE, but expressed that they would walk in order to have some type of physical activity: “I still walked like two or three [days]. I would say about 45 min to an hour,” (P10 MEG). Similarly to the in-person training, women in MEG preferred to have phone call conversations versus the text messages that were less interactive, less customized towards them, and were perceived as spam mail by some: “I read the text message, but just as soon as that came into my mind, it was gone. Her calls come in at a time where I’m not busy and I have time to focus on our conversation. Retaining the information or being able to prioritize what we’re saying may be the difference” (P8 MEG).

Women in SEG mentioned performing RE less frequently than women in MEG. Some participants would supplement a day of RE for cardio and others switched to performing cardio or other group exercises for the majority of their workouts. For example, “On my own I tried to make it to the gym at least once a week, but I don’t do as much strength training as much as I do cardio,” (P29 SEG) and, “I just decided to walk in the mornings from time to time,” (P15 SEG). Women in this group also felt that they would have benefited from access to their trainers to be able to discuss any challenges they were having. The removal of their trainer took away an accountability partner which led to lower levels of activity than the supervised training. Participants mentioned the desire for some form of communication from the study personnel in the following comments: “I don’t know if it’s a check in text saying, ‘hey, workout this week’, or maybe a partner or another person in the program that you can like partner up with afterwards, to help keep it going,” (P1 SEG) and, “Maybe being able to text if I had a question about something,” (P25 SEG).

Although the gym facility used during the supervised workouts was favorable to the women in the study, both groups stated that during unsupervised RE, they preferred to work out in their apartment gyms. Their gyms in their neighborhoods also provided a small and comfortable setting, but was more conveniently located than the gym facility used in the study: “My apartment complex is also less crowded like the gym [used during supervised sessions]. I don’t have to worry about machines being in use,” (P20 SEG); “I have a gym in my apartment complex, and [study’s gym facility] is like 15 min from my house. So I just didn’t want to make the drive,” (P17 SEG).

## 4. Discussion

Findings from this study support that a culturally adapted RE intervention specifically designed for young Black women was both feasible and acceptable during supervised RE, as evidenced by recruitment, retention, consent rates, questionnaire/survey completion, in-person adherence, and qualitative data, but was notably less successful when participants transitioned to unsupervised, independent RE. Particularly, there were salient themes regarding the importance of the program’s dedicated focus on Black women, which impacted both participants’ motivation to join as well as perceived comfort and relatedness during the intervention. Of note, findings indicate that the Black Women are F.I.R.E. intervention successfully promoted adherence to supervised RE, but that additional modifications to the protocol are required to enhance adherence rates during the transition to unsupervised RE. Key themes that emerged which can inform future iterations include the importance of continued accountability and support coupled with a desire for two-way text messaging or a more comprehensive mHealth platform to facilitate a more dynamic and tailored user experience during the unsupervised phase.

## 5. Intervention Feasibility 

Recruitment rates for this study were relatively high (97.2%) in comparison to previous exercise interventions including Black women and adults. For instance, recruitment rates for several studies were 22% [54], 89% [45], and 74.1% [4]; however, all were online, aerobic-based studies. For our study, overall retention rates were 90.0% at 12-week post-test and 86.7% at 24-week follow-up which are comparable to recent exercise interventions in Black women that were conducted in-person: 77.4% [61], 84.0% [44], 90.6% [62]. Historically, recruitment and retention have been challenging in interventions with Black or African American adults due to competing obligations, transportation issues, health complications, time constraints, and fear of participation given the historical events concerning this population [63,64,65,66]. Marginalized ethnic groups in the U.S. that participate in clinical trials typically report moderate levels of recruitment (64.0%) and retention rates (71.0%) [63]; therefore, even with the loss of several participants, the current study finds the feasibility of conducting a RE intervention in young Black women without children to be satisfactory.

The limited number of studies in Black women that have utilized RE were all in conjunction with aerobic exercise [24,26,27,28,29,30,31]. Furthermore only 50.0% reported adherence rates, none of which isolated RE-specific outcomes [26,27,28,29]. Despite this reporting gap, general adherence across these programs ranged from 70.0–93.0% in the experimental groups over the course of 12–24 weeks of supervised exercise, positioning the adherence rates of the current study at the upper end of the spectrum (MEG: 93.9% and SEG: 88.8%).

Several studies have assessed RE over longer timelines in cohorts of entirely [67,68], or predominantly women [33,69]. In those studies, supervised RE phases ranging from 3–9 months yielded high adherence rates (~95–97%) [69] [67]; however, independent participation declined substantially during unsupervised phases, with adherence rates dropping to roughly 24–75% across 12 weeks to 9 months [33,67,68,69]. Most studies defined adherence as two days/week [33,67,69] with the exception of one (three days/week) [68]. It is notable that these studies had longer supervised adherence periods and were conducted in middle-aged and older adult populations, which typically have higher adherence rates than younger adults [67,70,71].

In their interview responses, women indicated that they were drawn to this study because it was deliberately designed for Black women, emphasizing the deep relationships built with their trainers. Participants noted developing a “sisterhood” and gaining a friend in their trainer as they were able to have conversations beyond the scope of the study. This bonding aligns closely with an established framework for culturally adapting exercise interventions for Black women, which emphasizes that integrating socio-cultural norms and non-biological kinship bonds can create safe and validating spaces for participants [72]. These feelings of connectedness and belongingness can spark deeper levels of comfort and motivation to complete an intervention [73]. While integrating culturally relevant adaptations appears to coincide with strong adherence rates in exercise interventions within Black adults (>70%) [26,27,28,29,43], further exploration is needed to understand best practices to continue fostering these connections while unsupervised.

Participants also stated that last-minute adjustments to session times with their trainers were helpful which could have impacted the high in-person adherence rates. This population of young women have high autonomy in their daily schedules which are subject to change frequently and sometimes on short notice as seen in this study. Women who exercise regularly within this age group have reported that their exercise sessions are not always at the same time of the day [34]. Planning and scheduling exercise workouts for the week can promote consistent and regular exercise [8], but a program that allows for adjustments in weekly schedules could be beneficial for women in this age group. Women also mentioned that the small gym environment created a more intimate and comfortable atmosphere, which made learning a new type of exercise more enjoyable. This feeling of comfort could have also contributed to their higher adherence rates.

In the current study, supervised adherence was similarly high across both groups, suggesting that the additional behavioral strategies from the SCT and SDT did not meaningfully increase adherence beyond the core program. During the unsupervised phase, adherence to two days/week was low in both experimental groups, but women in MEG had a larger percentage of women who participated in at least one day per week of RE than the women in SEG (MEG: 64.3%; SEG: 38.5%). This could be attributed to the addition of continued contact with the study staff via scheduled text messages and phone calls while unsupervised, as women explicitly stated during their interviews that these conversations increased their motivation to work out. Although most of the women were completing one day of RE while unsupervised, our study methods proved to be insufficient for meeting the targeted national recommendations for muscle-strengthening as indicated by the low adherence rates to two or more days of unsupervised RE.

Women in MEG discussed their dissatisfaction with one-way text messages and desire for more tailored feedback; and participants in SEG, who had no contact with their trainers beyond in-person sessions, revealed the need for a greater level of accountability during unsupervised training. A long-term RE intervention study by Winett and colleagues demonstrated that two-way communication via phone calls with the participants while unsupervised resulted in adherence rates greater than 50% after 12 months of being unsupervised, thereby further underscoring the importance of ongoing, two-way contact from the study staff. Results from the current study suggest that one-way automated text messages are an insufficient modality for long-term adherence to unsupervised RE.

Participants in MEG explicitly valued personalized phone calls over automated texts, which were described as easily ignorable and impersonal. This preference highlights the need for more advanced features such as two-way communication and personalized feedback to provide accountability and support for ongoing RE. This is critical as RE requires a high level of self-regulation, evaluation, and affirmations regarding correct form and technique. Therefore, a more dynamic mHealth platform may be required to meet the needs of the participants to foster greater support for long-term, unsupervised RE adherence. Black women are willing to use mHealth strategies to engage in research [52], and those who have used mobile apps to promote better health habits have demonstrated high system usability rates of ~71.75 [74,75] (average = 68) [76], consistent engagement [35], and overall satisfactory rates with the component of the apps [74,77]. It is important to note that there have not been any mobile apps aimed to promote RE in Black women in the literature [57], emphasizing the demand for mobile apps to encompass exercise promotion beyond aerobic activities. Future studies should identify specific features, delivery mechanisms, and cultural adaptations for a mobile app desired by young Black women to help create sustained and interactive communication during unsupervised RE.

## 6. Intervention Acceptability

Despite women entering the intervention with preconceived feelings of intimidation and unfamiliarity with RE, in follow-up interviews, women spoke of a new sense of understanding, mastery, and comfortability with performing RE. Based on previous findings, it is normal for women to feel fearful or hesitant to perform RE due to lack of opportunities to learn, stigma surrounding RE being for men only, fear of developing a “manly” figure, and feeling out of place in strength areas of gyms [78]. Women in the current study expressed similar sentiments in addition to fear of injuring oneself. However, by the end of the study, women were more confident in their performance and even expressed a new liking for RE, which has also been conveyed by participants in other studies utilizing RE [43,79]. More findings are emerging in the literature that support that women are performing RE more regularly, [33,67,68,69,80] and some even prefer it as their primary mode of exercise [8], further underscoring the necessity to introduce and educate more women about RE.

Women were restricted to RE only during the supervised portion of the study, but once restrictions were lifted, they expanded their routines to include various forms of cardio either replacing RE or adding it to their current routines. Young women who exercise regularly who prefer cardio, such as walking or running, stated this preference due to its convenience [34]. Additionally, during this time, women were more likely to perform RE in their apartment gyms versus the community fitness center where they trained and still had access due to ease of access. Both convenience and accessibility are important considerations for designing future interventions for young Black women as both demonstrate being drivers of performing RE.

### Strengths and Limitations

This study is one of the first to assess the implementation of a culturally adapted behavioral intervention with a focus on RE in young Black women. Moreover, it provides data regarding long-term adherence to an exercise program which has been underreported in studies in Black women. However, some limitations must be addressed. The study used a simple coin toss for randomization; although this is a valid method of generating random assignment, it does not incorporate formal allocation concealment procedures. Another major limitation is the reliance on self-reported adherence rates during unsupervised RE, risking the chance of inaccurate estimates. Wearable devices have not been deemed to produce accurate or reliable measures of RE [81]. The authors chose not to use check-in scans at the facility used during supervised RE due to the possibility of participants using alternative locations.

Furthermore, generalizability is limited by our sample demographic, which consisted largely of young, educated, single college students without children. While this demographic experienced fewer scheduling and caregiving constraints, their adherence may have been affected by disruptions due to academic winter and summer breaks that altered their living situations and removed access to the fitness center provided during the study. A crucial point to highlight is the study dropouts were primarily due to childcare obligations. These attrition patterns underscore that caregiver responsibilities present unique structural barriers to RE participation and adherence. This, along with low adherence rates during unsupervised RE, points to the need for future, larger-scale trials to consider providing more flexible workout options (e.g., home-based) to accommodate women’s varying schedules and access to fitness facilities. Taken together, our findings may not be fully generalizable to Black women facing more severe socio-economic constraints, rigid work/school schedules, or familial obligations, or lacking access to fitness facilities.

Lastly, while semi-structured interviews were relatively brief, potentially limiting the opportunities to gather more in-depth responses, the number and the systematic analysis of the interviews support the authors’ beliefs that data saturation was reached based on no new information emerging and the findings accurately reflect the participant’s experiences.

## 7. Conclusions

This study highlights that culturally adapted strategies can enhance recruitment, in-person adherence, retention, and encouragement for supervised RE amongst inactive young Black women. While mobile support did not meet the expectations of the authors in this study nor were significant differences found in adherence rates between the two groups during supervised or unsupervised RE, responses from women in MEG underscored the need for more interactive mobile platforms that foster relatable and supportive experiences with their trainers. Overall, these findings present a significant and innovative approach to promoting health and targeting supervised exercise adherence which could potentially prevent and mitigate cardiometabolic disease risk factors in Black women. Future interventions should focus on refining mHealth components and enhancing communication between participants and their trainers to potentially strengthen adherence outcomes.

## Figures and Tables

**Table 1:** Weekly Topics and Theoretical Constructs for the MEG.

Week	Topic	Theoretical Concept
Week 1	Exercise safety, proper technique, and form	Competence (SDT)
Week 2	National PA recommendations, benefits of exercise, and basic principles of resistance training	Competence (SDT)
Weeks 3–5	Setting short- and long-term goals, self-monitoring behavior, strategies for managing time/schedule, common barriers to exercise for Black women	Goal setting, self-monitoring, time management (SCT)
Weeks 6–8	Recognizing and seeking social support, acknowledging accomplishments of goals, self-reflection on helpful strategies, providing autonomy support	Social support, self-monitoring, reinforcements (SCT), autonomy (SDT)
Weeks 9–11	Assessing comfortability with exercises, revisit goal setting, strategies to overcome barriers	Competence (SDT), self-monitoring, reinforcement, goal setting, relapse prevention (SCT)
Week 14	Reminder of goals, importance of tracking exercise behavior and setting reminders for exercise sessions	Goal-setting, self-monitoring, and time management (SCT)
Week 16	Acknowledgement of successes and setbacks	Reinforcements, relapse prevention (SCT)
Week 18	Reminder of eliciting social support and utilizing in-person and virtual support	Social support (SCT)
Week 20	Acknowledgement of successes and setbacks, external support from trainer	Reinforcements (SCT)
Week 22	Acknowledgment of internal feelings towards exercise and reminder of exercise goals	Goal-setting, reinforcements (SCT)

Notes: SDT = Self-Determination Theory; SCT = Social Cognitive Theory.

**Table 2:** Participant Demographics.

	Total (*n* = 27) Mean (SD) or %	MEG (*n* = 14) Mean (SD) or %	SEG (*n* = 13) Mean (SD) or %	*p*-Value	ƞ^2^
Age (years)	24.67 (3.77)	23.29 (3.77)	26.15 (3.29)	0.046	0.150
Marital Status				0.675	0.007
Single	88.9	85.7	92.3		
Married	0.0	0.0	0.0		
Cohabitating	7.4	7.1	7.7		
Divorced	3.7	7.1	0.0		
Children					
Zero	100.0	100.0	100.0		
Education				0.208	0.063
High School	3.7	7.1	0.0		
Some College	25.9	35.7	15.4		
Bachelor’s Degree	29.6	21.4	38.5		
Grad/Professional Degree	40.7	35.7	46.2		
Employment				0.089	0.111
Not employed	29.6	50.0	7.7		
Yes, <30 h	55.6	35.7	76.9		
Yes, ≥30 h	14.8	14.3	15.4		
Income				0.166	0.075
<$29,999	77.8	92.9	61.5		
$30,000–49,999	14.8	0.0	30.8		
$50,000–74,999	3.7	7.1	0.0		
$75,000–99,999	3.7	0.0	7.7		

Notes: Results of a multivariate analysis of variance; SD = standard deviation; α = 0.05.

**Table 3:** Qualitative Responses for the Acceptability of Supervised and Unsupervised Resistance Exercise Experiences.

12-Week Post-Test (MEG: *n* = 14; SEG: *n* = 13)	
Theme 1: Pre-study Perceptions	
Motivation to join	
Geared towards Black women Relatability to trainer Health Increase RE knowledge	
Perception of RE	
Intimidating Fear of bulky/manly figure	
Theme 2: Experience with Supervised Resistance Exercise	
Experience with RE Mental benefits: accomplished, stress relief, enjoyment Challenging More knowledgeable and confident in skills RE less intimidating	
Program Structure Small gym setting Accountability of trainer Flexibility of scheduling	
Interaction with Trainer Encouraging Developed friendship/sisterhood	
Weekly Topics (MEG Only) Preference for in-person discussions Less attentive to text messages	
Theme 3: Experience with Unsupervised Resistance Exercise	
24-week Follow-up MEG (*n* = 14)	24-week Follow-up SEG (*n* = 11)
Exercise Regimen ≥1 day of RE + cardio Increased activity after calls Preference for apartment gyms > Community Fitness Center	Exercise Regimen Less frequent RE, occasional cardio Preference for apartment gyms > Community Fitness Center
Mobile Experience Preference for phone calls Unlikely to read text messages	Limitations of Study No access to trainer Request for mobile check-ins

Notes: RE = resistance exercise.

## Data Availability

The datasets used and analyzed during the current study are available from the corresponding author on reasonable request.
